# GnRH Agonist and hCG (Dual Trigger) Versus hCG Trigger for Final Oocyte Maturation in Expected Normal Responders With a High Immature Oocyte Rate: Study Protocol for a Randomized, Superiority, Parallel Group, Controlled Trial

**DOI:** 10.3389/fendo.2022.831859

**Published:** 2022-03-28

**Authors:** Meng-Han Yan, Jing-Xian Cao, Jin-Wei Hou, Wen-Jing Jiang, Dan-Dan Wang, Zhen-Gao Sun, Jing-Yan Song

**Affiliations:** ^1^ The College of Traditional Chinese Medicine, Shandong University of Traditional Chinese Medicine, Jinan, China; ^2^ The First Clinical College, Shandong University of Traditional Chinese Medicine, Jinan, China; ^3^ Reproductive Center of Integrated Medicine, The Affiliated Hospital of Shandong University of Traditional Chinese Medicine, Jinan, China

**Keywords:** *in vitro* fertilization/intracytoplasmic sperm injection, normal ovarian responder, dual trigger, gonadotropin releasing hormone agonist, human chorionic gonadotropin

## Abstract

**Introduction:**

The choice of trigger drug for the controlled ovarian hyperstimulation (COH) protocol correlates with the outcome of *in vitro* fertilization/intracytoplasmic sperm injection embryo transfer (IVF/ICSI-ET). The co-administration of gonadotropin releasing hormone agonist (GnRH-a) and human chorionic gonadotropin (hCG), i.e., dual trigger, for final oocyte maturation, has received much attention in recent years. This trial was designed to determine whether a dual trigger approach by lengthening the time between trigger and ovum pick-up (OPU) improves the quantity and quality of mature oocytes/top-quality embryos and pregnancy outcomes in expected normal responders with a high immature oocyte rate.

**Methods and Analysis:**

We propose a study at the Affiliated Hospital of Shandong University of Chinese Medicine. A total of 90 individuals undergoing COH use a fixed GnRH antagonist protocol. They will be assigned randomly into two groups according to the trigger method and timing: recombinant hCG (6500 IU) will be injected only 36 hours before OPU for final oocyte maturation (hCG-only trigger); co-administration of GnRH-a and hCG for final oocyte maturation, 40 and 34 hours prior to OPU, respectively (Dual trigger). The primary outcome is metaphase-II (MII) oocytes rate. Secondary outcomes are number of oocytes retrieved, fertilization rate, top-quality embryos rate, blastula formation rate, embryo implantation rate, clinical pregnancy rate, miscarriage rate, live birth rate, cumulative pregnancy/live birth rates, and ovarian hyperstimulation syndrome (OHSS) rate.

**Ethics and Dissemination:**

The reproductive ethics committee of the Affiliated Hospital of Shandong University of Traditional Chinese Medicine certified this study (Identifier: SDUTCM/2021.7.26) as ethical. All individuals will sign written informed consent. All data and biological samples will be protected according to law. The results of this study will be disseminated in a peer-reviewed scientific journal.

**Clinical Trial Registration:**

[chictr.gov.cn], identifier [ChiCTR2100049292].

## Highlights

1.This is the first randomized controlled trial comparing hCG-only trigger and dual trigger (co-administration of GnRH-a and hCG, 40 and 34 hours prior to OPU, respectively) among normal ovarian responders with ≥ 50% immature oocytes in a previous IVF cycle triggered with hCG.

2.A prospective, randomized, superiority, parallel, controlled study aims to determine if co-administration of GnRH-a and standard dosage hCG trigger optimizes oocyte maturation, embryo quality, and pregnancy outcomes in normal responders when compared to hCG-only trigger.

3.Our findings will shed new light on IVF trigger approaches for infertile women with high immature oocyte rates.

## Introduction

At the moment, IVF/ICSI-ET is one of the most widely used techniques for addressing infertility. In order to obtain the ideal number and quality of oocytes, COH program came into being. hCG is usually used as a substitute for endogenous luteinizing hormone (LH) surge at the end of COH to induce follicular maturation and the continuation of meiosis. Nonetheless, because of the prolonged luteinization, using hCG alone as a trigger raises the risk of OHSS (1).. With the steady expansion of GnRH antagonist (GnRH-ant) use in clinical practice, a growing number of reproductive specialists are recommending GnRH-a as trigger medicines. GnRH-ant binding to the pituitary GnRH receptor is reversible. After GnRH-a injection, the receptor can dissociate from GnRH-ant and be activated by GnRH-a, allowing the acute release of endogenous LH and follicle stimulating hormone (FSH), i.e., flare up action. In comparison to spontaneous ovulation, the total quantity of endogenous gonadotropins (Gn) generated by GnRH-a triggering is lower and of shorter duration, which may contribute to the lower risk of OHSS. Nevertheless, GnRH-a triggers decreased pregnancy and live birth rates in fresh embryo transfer cycles, as well as higher risks of early miscarriage, requiring considerable luteal phase support (2, 3).

Given that fresh embryo transfer is the preferred option of assisted reproduction, oocyte maturation and ovulation induction by co-administration of GnRH-a and hCG, the dual trigger, have attracted much attention recently. In terms of number of oocytes retrieved, number of MII oocytes, fertilisation rate, top-quality embryos rate, and embryo implantation rate, a meta-analysis found that dual trigger was more advantageous but not statistically different from hCG trigger (4). Dual trigger is available in two ways (5, 6): Concomitant use GnRH-a and low-dose hCG (1000-2500 IU) as trigger drugs 35-37 hours prior to OPU reduces the risk of OHSS in high responders (7). And it can compensate for luteal insufficiency in GnRH-a trigger, which is associated with comparable or even higher pregnancy rates than hCG trigger (6–8)]. Furthermore, combining low-dose hCG with GnRH-a (dual trigger) to induce final oocyte maturation improved the number of oocyte retrieval in poor responders without raising the risk of clinically severe OHSS (9). Another alternative dual trigger approach is co-administration of GnRH-a and hCG for final oocyte maturation, 40 and 34 hours prior to OPU, respectively. Beck-Fruchter R et al. used this trigger to prolong the period between trigger and OPU to successfully obtain mature oocytes and achieve pregnancy, delivery in a patient with empty follicle syndrome (EFS) (10). According to recent findings, normal responders who had a lower rate of mature oocytes (less than 50%) shown a significant increase in the number of oocytes and transferable embryos obtained with this dual trigger method, as well as in the proportion of oocytes obtained to the number of preovulatory follicles, but the pregnancy rates were not conclusive (11, 12). Co-administration of GnRH-a and hCG 40 and 34 hours prior to OPU may therefore be considered novel therapeutic options for patients with poor mature oocyte rates. Additionally, Haas J et al. discovered that decreased conexin43 expression, in combination with enhanced epiregulin and amphiregulin expression, in the GCs of patients undergoing dual trigger may account for the better oocyte and embryo quality (13).

Although the results showed some benefit in terms of number of oocytes retrieved and MII oocytes using dual trigger in a prospective study involving all IVF/ICSI patients, there was no statistical difference. As a result, the population for whom the dual trigger strategy would be appropriate should be clarified further (14). Dual trigger has been shown to enhance oocyte maturation and improve top-quality embryo, pregnancy, and live birth rates in individuals with ovarian hypo-responsiveness (15, 16). Furthermore, a recent meta-analysis by Orvieto R shown that dual trigger increased the number of oocytes retrieved, the number of MII oocytes retrieved, and the number of high-quality embryos obtained in patients with poor maturation rates after prior hCG trigger cycles (7). In normal ovarian responders, the findings revealed that using GnRH-a and hCG simultaneously (dual trigger) substantially improved oocyte maturation in patients with poor oocyte maturation rates in hCG trigger cycles (17). Similar results were obtained in the study by Fabris AM and his colleagues (18). This suggests that when the oocyte maturation rate following a standard hCG trigger is less than 50%, the way GnRH-a combined with hCG to ultimately induce oocyte maturation should be considered in the next COH cycle. Nevertheless, there is increasing evidence, albeit in part controversial, that a dual trigger approach using both GnRH-a and hCG increases the number of MII oocytes, 2PN zygotes, transferable embryos, and pregnancy rates in normal ovarian responders (19–23). Yet, in individuals who have had a previous hCG trigger cycle, the benefit of trigger method utilizing GnRH-a and standard dosage hCG 40 and 34 hours prior to OPU, respectively, on oocyte retrieval, MII oocyte, transferrable embryo, and other reproductive outcomes remains unknown.

Trigger methods are related to the number of oocytes retrieved, mature oocytes, 2PN fertilisation rate, number of top-quality embryos, embryo implantation rate, clinical pregnancy rate, miscarriage rate, ongoing pregnancy rate, live birth rate, etc. The acquisition of mature oocytes is required prior to initiating IVF-ET. Obtaining higher-quality embryos is critical for IVF-ET success. The ultimate aim of IVF-ET is to maintain pregnancy and deliver a live baby. Therefore, a comprehensive evaluation of the patient situation and individualized formulation of the appropriate trigger method and trigger dosage are necessary. Previous studies have been compromised by sample size, data statistics, or technique, and debate has persisted about the study’s conclusions. The study is a prospective randomized superiority parallel controlled trial, we aimed to investigate whether co-administration of GnRH-a and standard dose hCG trigger could improve oocyte maturation, embryo quality and pregnancy outcomes in normal responders with poor oocyte maturation rates by prolonging the time between trigger and oocyte retrieval.

## Methods and Analysis

### Study Design

**Figure 1:**
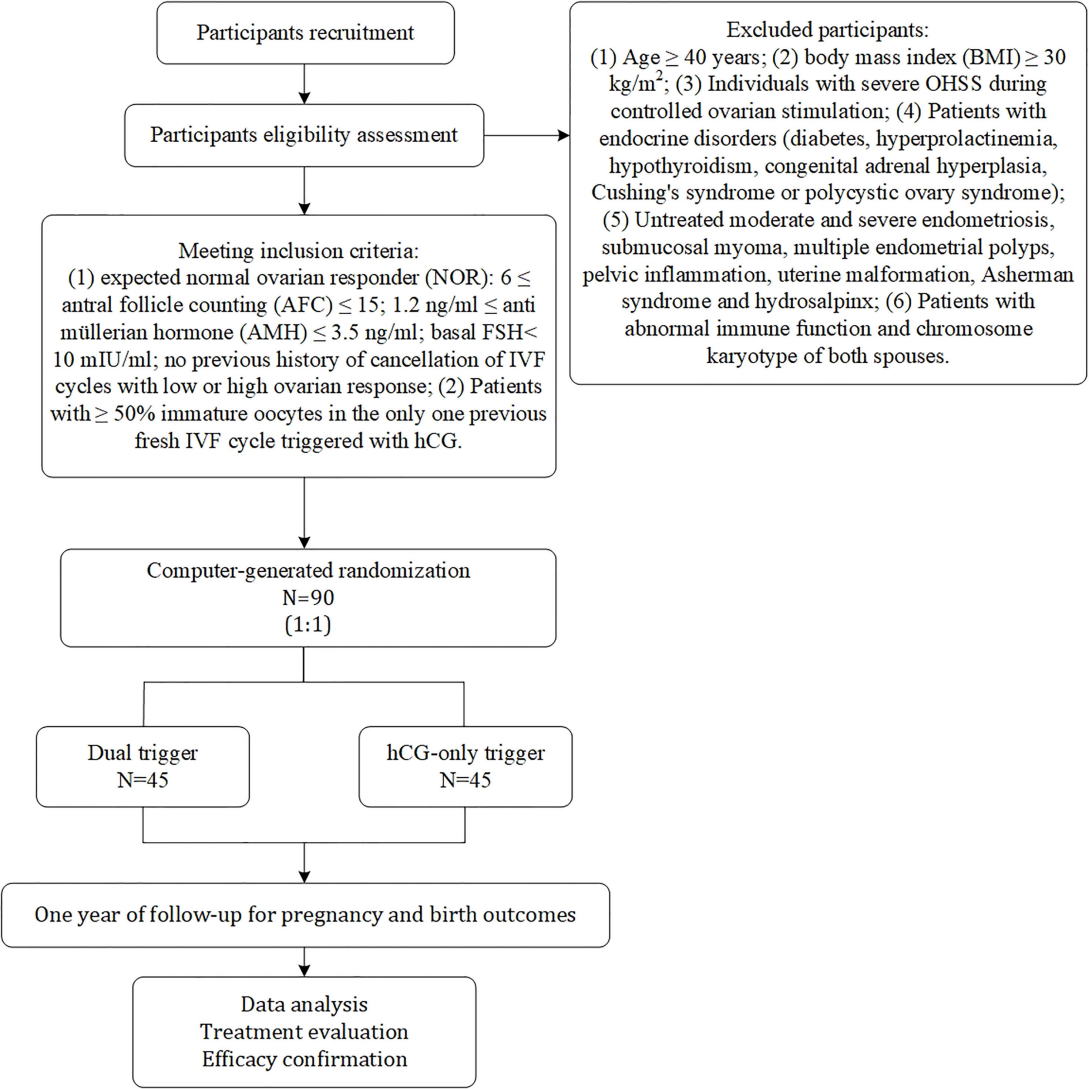
Flowchart showing the study process.

The study is a prospective, randomized, superiority, parallel, controlled trial. Enrolment of patients with normal ovarian response undergoing IVF/ICSI is expected to begin in July 2021 and continue until July 2023 at the reproductive centre of integrative medicine, the affiliated hospital of Shandong University of Traditional Chinese Medicine. We adhered to the SPIRIT recommendations when drafting this protocol (24). The flow chart for this study is shown in **Figure 1**, and the study process schedule is shown in **Table 1**.

**Table 1:** Overview of study visits.

	Enrolment Baseline	COH phase	hCG trigger	Dual trigger	Three days after OPU	Embryo transfer	Pregnancy testing	Follow-up 1 year
Information and counselling	X							
Signing of informed consent	X							
Treatment related data collection		X	X	X	X	X	X	X
Randomization			X	X	X	X		
Transvaginal ultrasound scan	X							X
Ovarian morphology UL scan	X	X	X	X	X	X		
Blood sample	X	X	X	X	X	X	X	

### Eligibility Criteria

#### Inclusion Criteria

(1) Expected normal ovarian responder (NOR): 6 ≤ antral follicle counting (AFC) ≤ 15; 1.2 ng/ml ≤ anti müllerian hormone (AMH) ≤ 3.5 ng/ml; basal FSH< 10 mIU/ml; no previous history of cancellation of IVF cycles with low or high ovarian response; (2) Patients with ≥ 50% immature oocytes in the only one previous fresh IVF cycle triggered with hCG; (3) In the previous IVF cycle, standard ovarian stimulation protocol was performed using a GnRH-ant protocol and triggered when two follicles were ≥ 18mm in diameter. 

#### Exclusion Criteria

(1) Age ≥ 40 years; (2) body mass index (BMI) ≥ 30 kg/m^2^; (3) Individuals with severe OHSS during ovarian stimulation; (4) Patients with endocrine disorders (diabetes, hyperprolactinemia, hypothyroidism, congenital adrenal hyperplasia, Cushing’s syndrome or polycystic ovary syndrome); (5) Untreated moderate and severe endometriosis, submucosal myoma, multiple endometrial polyps, pelvic inflammation, uterine malformation, Asherman syndrome and hydrosalpinx; (6) Patients with abnormal immune function and chromosome karyotype of both spouses.

#### Dropout Criteria

Participants who do not fulfil the inclusion criteria, do not adhere to standard treatment procedures, or refuse to sign an informed consent form as a result of a departure from the clinical trial protocol should be eliminated after enrolment. Participants that fulfilled the inclusion criteria but were dislodged from the clinical trial for various reasons, including two instances when the subject withdrew on their own and when the investigator noticed the subject had withdrawn.

### Study Case Discontinuation

Subjects in the study who experience serious adverse reactions, special physiological reactions, other unexpected events, or who are not suitable to continue participating in the trial will be terminated from the trial. Urgent measures will be taken during the study if the subject has a serious complication or if the condition worsens.

### Study Population and Recruitment

This trial will begin to recruit patients with infertility. Once the need for IVF/ICSI was determined and inclusion and exclusion criteria were met, patients were given detailed written information about the trial protocol. Approximately one week later, patients will be approached by the investigator and written informed consent will be obtained at the infertility clinic visit. Baseline blood tests will be performed and basic demographic information including infertility aetiology will be recorded. The patients were then randomly assigned to one of the study groups by the investigators and received the trial regimen.

### Interventions

#### Ovarian Stimulation Protocol

Standard COH protocol with gonadotrophins will be performed using a GnRH-ant protocol. Furthermore, a fixed dosage of GnRH-ant (0.25mg, Cetrorelix; Merck Serono, Darmstadt, Germany) will be used together with 112.5-225 IU/day of recombinant FSH (600IU, Puregon, Merck Sharp & Dohme B.V., Haarlem, Netherlands). Patients will initiate ovarian stimulation treatment with Gn starting on day 3 of the menstrual cycle, the starting dose of Gn will be based primarily on age and BMI, and determined by the patient’s attending physician prior to participation in the study. Subjects will undergo serial transvaginal ultrasound follicle measurements and monitoring of serum oestradiol (E_2_), progesterone (P_4_), and LH levels during the IVF/ICSI cycle to dynamically measured to evaluate the response of ovary, the dose of Gn will be adjusted according to the subject’s response. GnRH-ant (0.25mg/d) will be added on day 5 of ovarian stimulation sustained to the trigger. Trigger when two follicles were ≥ 18mm in diameter or when three follicles were ≥ 17mm in diameter. The following trigger patterns will be used in two groups: (1) hCG-only trigger: injection of recombinant hCG (6500 IU, Ovidrel, European Serono, France) 36 hours before OPU; (2) Dual trigger: co-administration of GnRH-a (Diphereline, 0.1mg, France, Epson) and hCG, 40 and 34 hours prior to OPU, respectively. If a patient has high-risk OHSS (more than 20 follicles over 10 mm in diameter) on the triggering day, a freeze-all strategy will be used. Oocyte retrieval will be performed by transvaginal puncture under transvaginal ultrasound guidance.

#### Embryo Culture

Oocytes will be enzymatically removed of cumulus cells and mature oocytes subjected to *in vitro* fertilization. Zoosperm will be cultured in pre-equilibrated embryo Petri dishes covered with mineral oil in an environment of 5.0% O_2_ and 5.6% CO_2_. The choice of embryos for vitrification will be expected to focus on the inclusion of no less than six blastomeres with ≤ 20% fragmentation. Embryos that presented a fragmentation rate between 20% and 50% will be transferred or vitrified only when they reach the 8-cell stage on Day 3. Day 5 blastocysts with a Gardner score of 3BB or higher were considered to be good quality and suitable for transfer or vitrification (25).

#### Luteal Phase Support Protocols

The decision on fresh or frozen embryo transfer will be made based on the patient’s membranes, serum P_4_ level, embryo score and personal requirements. Routine luteal phase support will be given after embryo transfer (ET), using intramuscular progesterone injection 40mg/day plus progesterone vaginal sustained release gel 90mg/day, discontinued from the day of ET until 10 weeks of gestation.

### Randomization and Blinding

On the trigger day, randomization will begin. Individuals will be assigned to one of two groups, A or B, in a 1:1 ratio by computer, using a random sequence of codes in the order of enrolment. The randomization scheme was entered into an online central randomization database (www.medresman.org). A dedicated data specialist will run the online randomization procedure, who will not be involved in patient recruiting or clinical care. The doctors will be notified of the allocation results *via* email after randomization. Allocation concealment will be guaranteed since the service will not divulge the allocation until after the randomization process, i.e. after the baseline visit. Due to the nature of the intervention, we will not blind the patients and doctors on the assigned groups. However, the trial outcome assessors will be blinded to the intervention.

### Blood Sample Collection

Blood sampling will be performed on the first day of the COH, during the ovarian stimulation, trigger day, oocyte retrieval day, 3 or 5 days after OPU and 14 days after fresh or frozen embryo transfer. Measurement of LH and FSH by time-resolved immunofluorescence. E_2_, P_4_ and hCG will be measured using commercial radioimmunoassay kits for serum measurement according to the manufacturer’s instructions. Freeze serum at -80 °C for use in research situations such as missing samples or analytical errors. If samples are not used, they will be destroyed within 5 years.

### Data Management, Informed Consent and Confidentiality

Data will be recorded electronically. An electronic copy will be stored in an encrypted file on the password protected computer. All data will be stored for 15 years. After that, electronic data will be formatted for removal. The organic samples will be stored in a safely locked temperature-controlled freezer in the centre that can only be used by authorized personnel until the end of the analysis. The sample and participant records do not contain any directly identifiable information. Additional biospecimens will not be retained for ancillary studies.

### Reproductive Outcomes

The primary outcome is MII oocytes rate (percentage of the number of mature oocytes to the number of oocytes retrieved).

The secondary outcomes are number of oocytes retrieved, fertilization rate (the proportion of oocytes that become fertilized), top quality embryos (TQE) rate (percentage of the number of TQE to total embryos obtained), blastula formation rate, embryo implantation rate (percentage of the number of gestational sac to the total transferred embryos), clinical pregnancy rate (percentage of participants with clinical pregnancy), miscarriage rate (percentage of participants with loss of a diagnosed clinical pregnancy before 12 weeks gestation), live birth rate (the proportion of a delivery of one or more living infants), cumulative pregnancy/live birth rates (percentage of participants with clinical pregnancy/live birth after one year of follow-up), severe OHSS rate (percentage of participants with OHSS). TQE was defined as seven or more blastomeres on day three, evenly sized blastomeres, and less than 20% fragmentation, whereas low quality embryos were characterized as anything else. On transvaginal ultrasonography, clinical pregnancy was defined as the appearance of a gestational sac and foetal heartbeat.

### Sample Size Estimation

This clinical study is a superiority, randomized parallel controlled trial. GnRH-a+hCG (dual trigger) in group A and hCG-only trigger in group B. MII oocytes rate will be the primary outcome measurement. According to previous data of our centre, the estimated rate of MII oocytes in group B was **40%**, and **70%** in group A after using GnRH-a (40h before OPU) + hCG (34h before OPU) dual trigger, with **standard deviations of 40%** for each group. Suppose that α=0.05 (two-sided) and β=0.10 (90% power), the sample size is calculated to be 40 individuals for each group by PASS 15.0 (NCSS, LLC. Kaysville, Utah, USA.). Assuming a dropout rate of 10%, the sample size 45 individuals for each group will be required. Ultimately, a total of 90 individuals will be recruited for this study.

### Data Statistical Analysis

All randomized individuals will be included in the analysis of the primary trial end point (intention-to-treat analysis). For analyses of other outcome variables, only subjects randomized to the study protocol will be included (per-protocol analysis). SPSS version 26.0 and R statistical package version 4.0.0 will be used to analyse all the data. The continuous data will be normalized using the two independent samples t-test and presented as mean ± standard deviation (Mean ± SD). If the distribution is not normal, use nonparametric tests, which are expressed as the median (interquartile range) [M (IQR)]. Categorical data will be presented as frequency (percentage) [n (%)] using chi square test and Fisher’s exact test for expected frequencies less than five. P < 0.05 indicates that the difference is statistically significant.

### Adverse Events and Data Safety and Monitoring

An independent data safety and monitoring committee will be established to monitor the occurrence of adverse events. During the trial, the principal investigator will be followed up by telephone at any time and subjects will provide details if any adverse events arise. Participants will also be interviewed on the day of oocyte retrieval to determine if any adverse events occur serious adverse events will be recorded separately and will be followed up until resolution.

### Concomitant Care

Standard concomitant treatments such as folic acid supplements will be allowed in addition to the trial protocols and inclusion/exclusion criteria listed above.

### Patient and Public Involvement

Women and their partners beginning ART therapy at the reproductive centre of integrative medicine, the affiliated hospital of Shandong University of Traditional Chinese Medicine, will be included in the study. We asked about their experience of assisted conception, the things they liked and disliked, and the potential difficulties or barriers to attending for treatment, which may affect randomization. Participant requirements for the transfer of one or two embryos may have influenced part of the results. All elements of this study (creation of the study topic, study design and study conduct, interpretation of findings, and final manuscript editing for publication) are conducted without the participation of patients or the general public. The findings will be allowed to share with participants by their physicians.

### Ethics and Dissemination

The study was approved by the reproductive ethics committee of the Affiliated Hospital of Shandong University of Traditional Chinese Medicine (Identifier: SDUTCM/2021.7.26). Any modifications to the protocol that may affect the management, design, conduct, or safety of the study require a formal amendment to the Committee. COH followed by pharmaceutical trigger will be performed according to the routine IVF procedure. Safety was considered high for trial participants. All participants will provide written informed consent. No additional financial costs were required for study participants other than transportation. The results of this study will be disseminated in a peer-reviewed scientific journal.

## Discussion

In IVF/ICSI technology, the use of a pharmaceutical trigger to drive the final maturation of the oocyte is a crucial step. The LH effect produced by natural cycle lasts for 48 hours. As a common pharmaceutical used as trigger, hCG plays a role in promoting the final maturation of oocytes instead of endogenous LH. But the effect of 10, 000 IU hCG is 20 times greater than that of endogenous LH and can last more than 120 hours (1). Although the effects of hCG are long-lasting and can promote the production of corpus luteum in favour of embryo implantation and ongoing pregnancy. But it is precisely because of its strong and sustained action that leads to an increased risk of OHSS during the hCG trigger cycle. GnRH-a trigger has the advantage of driving the production of endogenous LH and FSH. LH and FSH act synergistically to promote oocyte final maturation and ovulation, which seems to be more favourable for oocyte quality. However, due to the effects of GnRH-a last only 24-36 h, leading to subsequent luteal insufficiency, they adversely affect pregnancy outcomes and live birth rates. Thus GnRH-a trigger still has certain limitations in the clinic.

The use of GnRH-a and a standard dose of hCG at 40 and 34 hours prior to oocyte retrieval was previously found to produce an elevated proportion of low mature oocytes compared to previous hCG trigger cycles and to successfully obtain mature oocytes in a patient with EFS. This study is a prospective, randomized, superiority-controlled trial to investigate whether dual trigger could improve reproductive outcomes in people with NOR, with the aim of identifying a more optimal trigger method. If this trigger method does improve IVF/ICSI outcomes, it may become a routine trigger method to boon more infertile couples. The subsequent results of this study, which may provide some data support for our next randomized controlled trial.

## Ethics Statement

The reproductive ethics committee of the Affiliated Hospital of Shandong University of Traditional Chinese Medicine certified this study (Identifier: SDUTCM/2021.7.26). The patients/participants provided their written informed consent to participate in this study.

## Author Contributions

J-YS conceived of the study. M-HY and J-YS responsible for data collection and thesis writing. J-XC, W-JJ, J-WH, and D-DW responsible for data curation and supervision. Z-GS responsible for the revision of articles and enrolment of patient. All authors have read and approved the manuscript.

## Funding

This study was funded by the National Natural Science Foundation of China (81874484).

## Author Disclaimer

The authors are fully responsible for the content of this manuscript, and the views and opinions described in the publication reflect solely those of the authors.

## Conflict of Interest

The authors declare that the research was conducted in the absence of any commercial or financial relationships that could be construed as a potential conflict of interest.

## Publisher’s Note

All claims expressed in this article are solely those of the authors and do not necessarily represent those of their affiliated organizations, or those of the publisher, the editors and the reviewers. Any product that may be evaluated in this article, or claim that may be made by its manufacturer, is not guaranteed or endorsed by the publisher.

## Abbreviation

COH, Controlled Ovarian Hyperstimulation; IVF/ICSI-ET, *In Vitro* Fertilization/Intracytoplasmic Sperm Injection Embryo Transfer; hCG, Human Chorionic Gonadotropin; OPU, Ovum Pick-Up; GnRH, Gonadotropin Releasing Hormone; GnRH-ant, Gonadotropin Releasing Hormone Antagonist; GnRH-a, Gonadotropin Releasing Hormone agonist; LH, Luteinizing Hormone; OHSS, Ovarian Hyperstimulation Syndrome; FSH, Follicle Stimulating Hormone; Gn, Gonadotropin; MII, Metaphase-II; NOR, Normal Ovarian Responder; AFC, Antral Follicle Counting; AMH, Anti Müllerian Hormone; BMI, Body Mass Index; E_2_, Oestradiol; P_4_, Progesterone; EFS, Empty Follicle Syndrome.
